# Risk of opioid misuse in chronic non-cancer pain in primary care patients - a cross sectional study

**DOI:** 10.1186/s12875-018-0775-9

**Published:** 2018-06-20

**Authors:** Johannes Maximilian Just, Linda Bingener, Markus Bleckwenn, Rieke Schnakenberg, Klaus Weckbecker

**Affiliations:** 1 Institute of General Practice and Family Medicine, Bonn University Clinic, Sigmund-Freud-Street 25, 53127 Bonn, Germany; 2 Department for Health Services Research, Carl von Ossietzky Universität Oldenburg, Post office box 2503, 26111 Oldenburg, Germany

**Keywords:** Opioid, Misuse, Addiction, Prescription drugs, Prevalence

## Abstract

**Background:**

Efforts to improve treatment of pain using opioids have to adequately take into account their therapeutic shortcomings which involve addictiveness. While there are no signs of an “opioid epidemic” in Germany similar to that in the US, there is little data on the prevalence of prescription opioid misuse and addiction. Therefore, our objective was to screen primary care patients on long-term opioid therapy for signs of misuse of prescription opioids.

**Methods:**

We recruited 15 GPs practices and asked all patients on long-term opioid therapy (> 6 months) to fill out a questionnaire including the “Current Opioid Misuse Measure” (COMM®), a self-report questionnaire. Patients with a malignant disease were excluded.

**Results:**

*N* = 91 patients participated in the study (response rate: 75.2%). A third (31.5%) showed a positive COMM® - Score which represents a high risk of aberrant drug behaviour. A positive COMM® - Score showed a statistically significant correlation with a lifetime diagnosis of depression and neck pain.

**Conclusions:**

While Germany does not face an “opioid eoidemic”, addictiveness of opioids should be considered when using them in chronic non-tumor pain. In our study population, almost every third patient was at risk and should therefore be followed up closely. Co-prevalence of depression is a significant issue and should always be screened for in patients with chronic pain, especially thus with aberrant drug behaviour.

## Background

Opioids are a cornerstone in the treatment of acute and chronic pain, still their therapeutic shortcoming have to be considered [1]. They involve risk of addiction, a narrow therapeutic ratio and lack of documented effectiveness in the treatment of several aspects of chronic non-cancer pain (CNCP) [2]. Misuse and addiction from prescription opioids is a serious public health issue in the US. The death toll has almost quadrupled in the 21st century, matching a similar increase in prescription rates [2, 3]. A meta-analysis calculated an average proportion of misuse between 21 and 29% (range, 95% confidence interval [CI]: 13–38%) for patients with CNCP [4]. Currently, significant effort is put into reversing these effects, including a new CDC guideline for “Prescribing Opioids for Chronic Pain” [2].

The increase of opioid prescription in the US is paralleled by an increase in all European countries [5]. A meta-analysis on medication misuse in the EU named prescription opioids as a main group of misuse but data on mortality directly linked to opioids does not exist in Germany [6]. The increase in opioid prescriptions in Germany is clearly less extreme than in the US. The percentage of persons with statutory health insurance who have been prescribed opioids at least once per year has increased from 3.3% in 2000 to 4.5% in 2010 [7]. An analysis of randomly selected claims records of 870,000 persons in a large German medical health insurance organization showed a pooled 1-year prevalence of abuse/addiction (defined as hospital stays related to addiction) of 0.008% in those on long-term opioid therapy (LTOT). Therefore, the authors concluded, that “there are no signals of an ‘opioid epidemic’ in Germany” [8].

Factors that may have contributed to the opioid epidemic in the US include higher doses of opioids per patient compared to other developed countries, a high individual health care cost burden, lesser regulatory restrictions for opioids and a “pro-profit” orientation of key elements of the health care system [9–11]. The German medical system on the contrary offers compulsory, high-quality health care with adequate regulatory restrictions for opioids including a ban on direct to consumer marketing by the pharmaceutical industry.

The lack of many society related contributing factors to opioid misuse in Germany is reassuring. Nevertheless addictive behaviour is a world-wide problem and more data on possible at-risk patients in Germany could help to keep rates of opioid misuse low. Therefore we conducted a study, screening primary care patients on LTOT for risk of misuse of prescription opioids, using a validated self-report measure [12].

## Methods

We conducted a cross sectional study at GP’s practices using the self-report questionnaire Current Opioid Misuse Measure (COMM®), adding items concerning the medical history as well as socio-economic information. COMM® Score results were the primary outcome criterion.

The COMM® Score is a self-report questionnaire for patients using opioids longer than six months. We used an existing German version of the COMM®. The questionnaire was translated from English to German by experienced translators using the “back-translation method”. Then, a team of bilingual addiction experts fine-tuned the questionnaire in order to make sure that the meaning and intent of the original items were preserved [13]. Later, it was pre-tested for comprehensibility and acceptability with five representative patients by our study group, using cognitive interviewing techniques.

In order to recruit GPs, we contacted all GPs in the greater Bonn area (> 100) via fax and telephone. We stopped recruiting after having reached the planned number of 15 GPs. A total of four GPs refused to participate due to their high workload.

All patients on LTOT (> 6 months) for CNCP, who entered the practice to collect their prescription, were asked to fill in the questionnaire by the front desk staff. The study period was three months, as it represents the maximum duration of one prescription in Germany, making sure we included all relevant patients. Those who did not want to participate were asked to fill in their age and sex to test for differences in participants and nonparticipants.

Inclusion criteria were LTOT (> 6 months) for CNCP and sufficient literacy. Excluded were patients with malignant disease, patients who could not collect their prescription themselves due to age or multimorbidity and patients on opioid-maintenance therapy.

The data management was performed at the Department of General Practice and Family Medicine in Bonn and included data entry, data validation by plausibility checks, frequency analyses and advanced statistics using IBM SPSS Statistics 22®. We used exploratory statistics and a multiple logistic regression model with a dichotomous dependent variable to describe data and to explore the relationship between dependent and independent variables. For comparison of participants and nonparticipants concerning age and sex, we used Student’s t-test and Pearson’s chi-squared test.

The anonymous survey received ethical approval by the Ethics Committee of the Medical Faculty of the University of Bonn (No. 243/16).

## Results

The inclusion criteria were met by 121 patients from 12 GPs practices, of which 91 completed the questionnaire (response rate: 75.2%). Participants mean COMM® - Score was 7.26 (min-max: 0–41).

On average, we received nine (min - max: 0–17) questionnaires per practice. One practice claimed they did not have any patients matching our criteria, two practices did not proceed with the study due to “work overload” and were therefore considered “drop-outs” (rate: 13%).

All of the non-participants agreed to have their age and sex documented. Non-participants differed slightly from participants concerning age and sex, without differences being statistically significant (Table 1).

**Table 1:** Age and sex distribution for participants and nonparticipants

Variable	All participants (*n* = 93)	Non participants (*n* = 28)	Difference significant
Age (mean)	69.70 (SD: 14.39; min/max: 25/93)	74.79 (SD: 14.06; min/max: 46/92)	No^a^
Sex: female	62.4%	60.7%	No^b^

^a^Student’s t-test (df: 119)

^b^Pearson’s chi-squared test (df: 2)

The mean age of participants was 69.70 years (SD 14.39), 62.4% were female. Most participants reported back and joint pain as the reason for taking opioid analgesics. The proportion of participants with a “high risk of aberrant drug behaviour” according to COMM® (meaning a score of nine or higher) was 31.5%. Of these patients, 64.3% had a high school education only. A statistically significant correlation with a positive COMM® - Score was present for the variables “lifetime diagnosis of depression” and “neck pain”. A detailed description of all variables and the connected odds ratio for a positive COMM®-Score is given in Table 2.

**Table 2:** Risk factors for positive COMM - Score (logistic regression analysis, *n* = 91, df = 14)

	CommScore positive	CommScore negative	OR (95% CI)	Sig.
N (%)	28 (31.5%)	65 (60.5%)	N/A	N/A
Gender, male	9 (32.1%)	25 (39.1%)	0.85 (0.25–2.87)	0.79
Age (mean (SD))	69.61 (17.2)	69.74 (13.2)	0.99 (0.95–1.04)	0.69
Headache	5 (17.9%)	6 (9.2%)	1.80 (0.27–12.19)	0.55
Back pain	23 (82.1%)	43 (66.2%)	1.45 (0.37–5.65)	0.60
Joint pain	17 (60.7%)	30 (46.2%)	2.70 (0.72–10.16)	0.14
Neck pain	10 (35.7%)	7 (10.8%)	9.23 (1.63–52.26)	0.01
Rheumatic pain	7 (25.0%)	11 (16.9%)	0.94 (0.20–4.53)	0.94
Postoperative pain	5 (17.9%)	10 (15.4%)	0.57 (0.12–2.75)	0.48
Other pain	4 (14.3%)	11 (16.9%)	0.28 (0.04–1.92)	0.19
Prior addiction diagnosis	2 (7.1%)	2 (3.1%)	1.34 (0.11–16.99)	0.82
Addiction diagnosis in family	3 (10.7%)	4 (6.2%)	5.55 (0.61–50.61)	0.13
Depression	17 (60.7%)	16 (25%)	6.84 (1.88–24.91)	0.004
Fear of Addiction	11 (39.3%)	18 (27.7%)	0.85 (0.24–3.06)	0.80
Education, high school or lower	18 (64.3%)	27 (41.5%)	3.13 (0.92–10.62)	0.07

N = absolute number of participants; (%) = percentage within variable COMM - Score; OR = Odds ratio; (95% CI) = 95% Confidence Interval for Odds ratio, Sig. = Significance of OR

We used a multiple regression model to control for statistically significant correlations between positive COMM® - Scores and the documented patient characteristics. The model was sound, showing a Nagelkerke’s Pseudo-R^2^ of 0.401. All tested variables are shown in Table 2.

## Discussion

Our study showed a high risk of aberrant drug behaviour in almost one third (31.5%) of the targeted patient group. With 75.2%, the response rate was good. Back and joint pain were the most commonly reported reasons for taking opioid painkillers. A statistically significant correlation with a positive COMM® - Score was present for the variables “lifetime diagnosis of depression” and “neck pain”.

The proportion of “at risk patients” seems rather high, regarding the assumption that there is no opioid epidemic in Germany. In this context it needs to be emphasized, that we screened for patients how are at risk of developing misuse only. So there probably is potential for an opioid epidemic in Germany, but positive physician- and society related factors might have prevented such a development.

The proportion of “at risk” patients may have been overestimated in our study. The COMM® - Score uses several questions that target signs of emotional volatility that might be present in depression as well as in opioid misuse. As depression is more prevalent in patients with CNCP, this might explain the high proportion of COMM® - Score positive patients in the sample. Additionally, this may also explain the high proportion of patients with depression within the group of COMM® - Score positive patients. More than half of COMM® - Score positive participants reported a history of a diagnosis of depression (60.7%) with a statistically significant correlation between a positive score and the presence of depression. The average lifetime prevalence of depression in Germany in contrast is 11.6%, the prevalence of depression in a large European chronic pain patient cohort was 21% [14, 15]. So while the methodology does not allow us to make a statement concerning causality, and there might be a bias towards over-diagnosing risk of misuse in patients with depression, the high prevalence of depression should be kept in mind when treating patients with opioid misuse.

Some known risk factors connected to the opioid epidemic from prior studies in the US were not found in our study population (e.g. young age, male sex). [16, 17]. So the particularly alarming increase in opioid misuse in young, male subjects in the US seems not to be an issue in our sample group [18]. Numbers for “prior addiction diagnosis” and “addiction diagnosis in relatives” were very small (*n* < 5) in our study, which explains non-significant results for these well described risk factors.

The statistically significant correlation of neck pain and a positive COMM® - Score in the logistic regression model is an interesting new finding. Psychosocial stress factors may have acted as a confounding factor, as they are contributing factors to both, substance abuse and neck pain [19–21]. Still, numbers are small and it would be interesting if our findings can be reproduced in larger samples for instance using secondary analysis of a large insurance database.

There are several further limitations to this study. It is difficult to control for bias that stems from different prescription patterns in doctors and we did only include patients from 12 different GPs practices. So we cannot claim that our sample is representative. Still they give a first hint at which proportion of opioid misuse in CNCP can be expected on a national level. The sample group of GP practices can be considered a convenience sample. While we did contact all GPs in the area, we did not randomly choose from those willing to participate. We did so to achieve higher numbers of participants but this could have led to a selection bias.

Generally, the Bonn area has a high socio-economic status, so we controlled for this factor using educational status as an indicator and tried to achieve socio-economic heterogeneity concerning the location of the participating practices. As shown in Table 2, there was no significant difference between COMM® - Score positive and negative patients in relation to their educational status. Furthermore, age adjusted average educational levels in Germany did not differ greatly when compared with the group of COMM® - Score positive patients (basic high school education or lower in COMM® - Score positive patients: 64.3%, age adjusted German average: 59.9%) [22]**.**

The COMM® is a reliable and valid screening tool to help detect current aberrant drug-related behaviour among chronic pain patients [12]. We used a thorough method of translation and German language adaptation. Data from China suggests that the COMM® shows satisfactory reliability and validity, despite the arguably high cultural gap between China and the US [23]. Still it is a shortcoming of this study, that we did not perform tests for internal consistency, test-retest reliability, exploratory factor analysis and confirmatory factor analysis.

## Conclusion

In summary, our study showed that 31.5% of patients with CNCP on LTOT in Germany might be at a high risk for aberrant drug behaviour. This does not signify that these patients are addicted yet, albeit their risk is probably increased. Considering opioids shortcomings (low therapeutic ratio, lack of documented effectiveness in the treatment of several aspects of chronic non-cancer pain), these at risk patients should be followed up regularly. Depression should always be screened for and treated in CNCP and the high co-incidence of addiction risk and depression should be acknowledged when doing so.

## Abbreviations

CNCP: Chronic non-cancer pain
COMM®: Current opioid misuse measure
LTOT: Long-term opioid therapy
